# Focal Spot and Wavefront Sensing of an X-Ray Free Electron laser using Ronchi shearing interferometry

**DOI:** 10.1038/s41598-017-13710-8

**Published:** 2017-10-20

**Authors:** Bob Nagler, Andrew Aquila, Sébastien Boutet, Eric C. Galtier, Akel Hashim, Mark S. Hunter, Mengning Liang, Anne E. Sakdinawat, Christian G. Schroer, Andreas Schropp, Matthew H. Seaberg, Frank Seiboth, Tim van Driel, Zhou Xing, Yanwei Liu, Hae Ja Lee

**Affiliations:** 1 SLAC National Accelerator Laboratory, 2575 Sand Hill Road, Menlo Park, CA 94025 USA; 2 Deutsches Elektronen-Synchrotron (DESY), Notkestrasse 85, D-22607 Hamburg, Germany; 3 Department Physik, Universität Hamburg, Luruper Chaussee 149, D-22761 Hamburg, Germany

## Abstract

The Linac Coherent Light Source (LCLS) is an X-ray source of unmatched brilliance, that is advancing many scientific fields at a rapid pace. The highest peak intensities that are routinely produced at LCLS take place at the Coherent X-ray Imaging (CXI) instrument, which can produce spotsize at the order of 100 nm, and such spotsizes and intensities are crucial for experiments ranging from coherent diffractive imaging, non-linear x-ray optics and high field physics, and single molecule imaging. Nevertheless, a full characterisation of this beam has up to now not been performed. In this paper we for the first time characterise this nanofocused beam in both phase and intensity using a Ronchi Shearing Interferometric technique. The method is fast, *in*-*situ*, uses a straightforward optimization algoritm, and is insensitive to spatial jitter.

## Experimental Description

The start of operations of Free electron laser both in the Extreme UV^1,2^ and in the hard X-ray regime^3–5^ has created sources of unmatched brilliance, that are advancing many scientific fields at a rapid pace (see^6,7^ and references therein). A complete focal characterization in both intensity and phase is of crucial importance in application such as coherent diffractive imaging^8^, non-linear x-ray optics and high field physics, and single molecule imaging where the highest X-ray intensities are sought.

A standard technique currently used involves evaluating the size of damage craters created by the focused X-ray beam in a target^9^. While this method has the advantage that it measure the whole intensity profile (i.e. both the coherent and incoherent part), it requires a time consuming post mortem analysis of many such imprints, is not an *in*-*situ* method, and has limited spatial resolution. Scanning coherent diffraction microscopy or ptychography has been successfully used to characterise a focused X-FEL beam^10^. However, ptychography requires a 2D motorized translation stage with approximately 10 nm resolution and a beam pointing stability of the same order, which is not always available. Alternatively, interferometric methods using shearing interferometry have been pursued both at Free electron laser facilities^11^ and at synchrotron facilities^12,13^. Here we present a method to fully determine the focus of an X-FEL using Ronchi shearing interferometry. Ronchi testing has a long history in characterizing the quality of focusing optics in optical wavelengths^14^. It has more recently been used to qualitatively evaluate the X-ray optics at both synchrotron facilities^15^ and at X-ray Free Electron Lasers^16^. In this paper we show a full characterization of the amplitude and phase of a nano-focused X-FEL beam using Ronchigrams.

**Figure 1:**
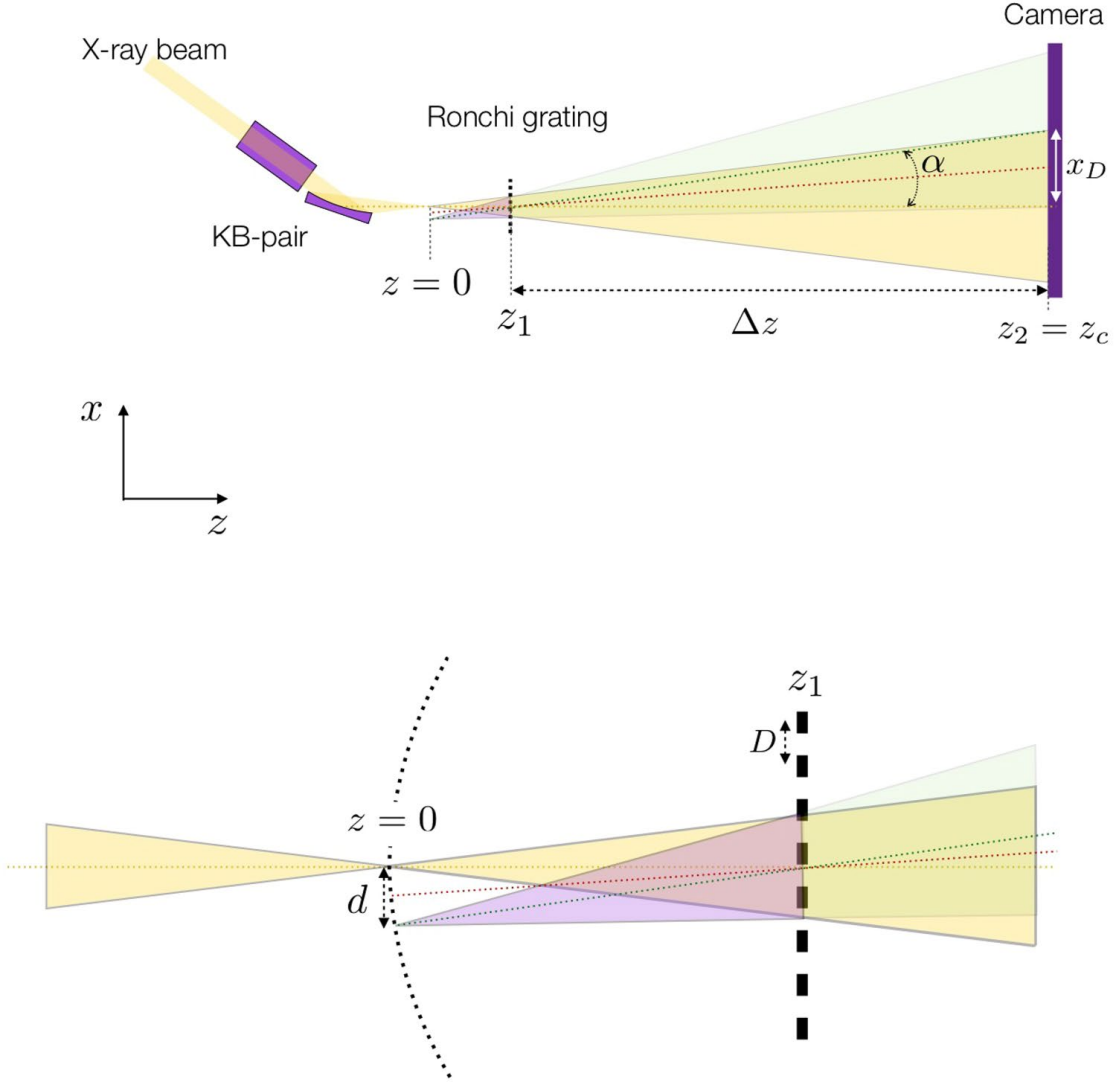
Conceptual sketch (not to scale) of the setup. The period of the Ronchi grating is chosen such that order +1 and −1 do not overlap, while maintaining as large an overlap between order 0 and the first orders. Only the 0 and +1 orders are shown for clarity.

The experiments were performed at the Coherent X-ray Imaging instrument (CXI)^17,18^ beamline at the Linac Coherent Light Source (LCLS). The CXI instrument has a pair of highly polished Kirkpatrick-Baez (KB) mirrors^19^ coated with Silicon Carbide^20^ to focus the beam to a theoretical minimal spot size of 90 nm by 150 nm^17,21^. In contrast to the beamlines that use Beryllium lenses to create a focus, it doesn’t suffer from chromatic abberation and its aperture is large enough to capture the full beam. Therefore, it routinely creates the highest peak X-ray intensities of the facility, estimated to be in the order of 1 × 10^20^ W/cm^2^. It has been used in many fluence dependent experiments such as the formation of hollow atoms^22^, anomalous nonlinear X-ray Compton scattering^23^, and radiation damage studies on protein microcrystals^24^. In the experiment presented here, the LCLS beam, with a photon energy of 7.2 keV was focused with the KB-mirror pair, which has focal lengths of 900 mm in the horizontal direction, and 500 mm in the vertical direction. A one dimensional diffraction grating, (i.e. the Ronchi target) is placed 9.3 mm downstream of the X-ray focal plane. An X-ray detector is placed 982 mm downstream of the Ronchi target. A conceptual sketch of the setup can be seen in Fig. 1.

Ronchi gratings were fabricated on 4 *μ*m thick polished diamond membranes (Diamond Materials GmbH). An 8 nm layer of Ti was evaporated on the diamond, and 150 nm hydrogen silsesquioxane (HSQ) resist was spun on top of the Ti. Electron beam lithography was performed using a 100 keV beam with doses ranging from 2200–2600 *μ*C/cm^2^. The gratings were then developed in 25% wt tetramethyl ammonium hydroxide (TMAH) for 100 seconds and rinsed with isopropyl alcohol and deionized water. Transfer of the HQ grating pattern into diamond was performed using reactive ion etching. A 15 second titanium etch using chlorine was used to remove the Ti layer. The diamond was then etched for 50 minutes using an O_2_/Ar plasma (33/17 sccm, 10 mTorr, RIE power = 100 W) until the etch depth reached 1.1 *μ*m. Using atomic layer deposition (ALD), 78 nm platinum was deposited conformally to fill the diamond gratings.

**Figure 2:**
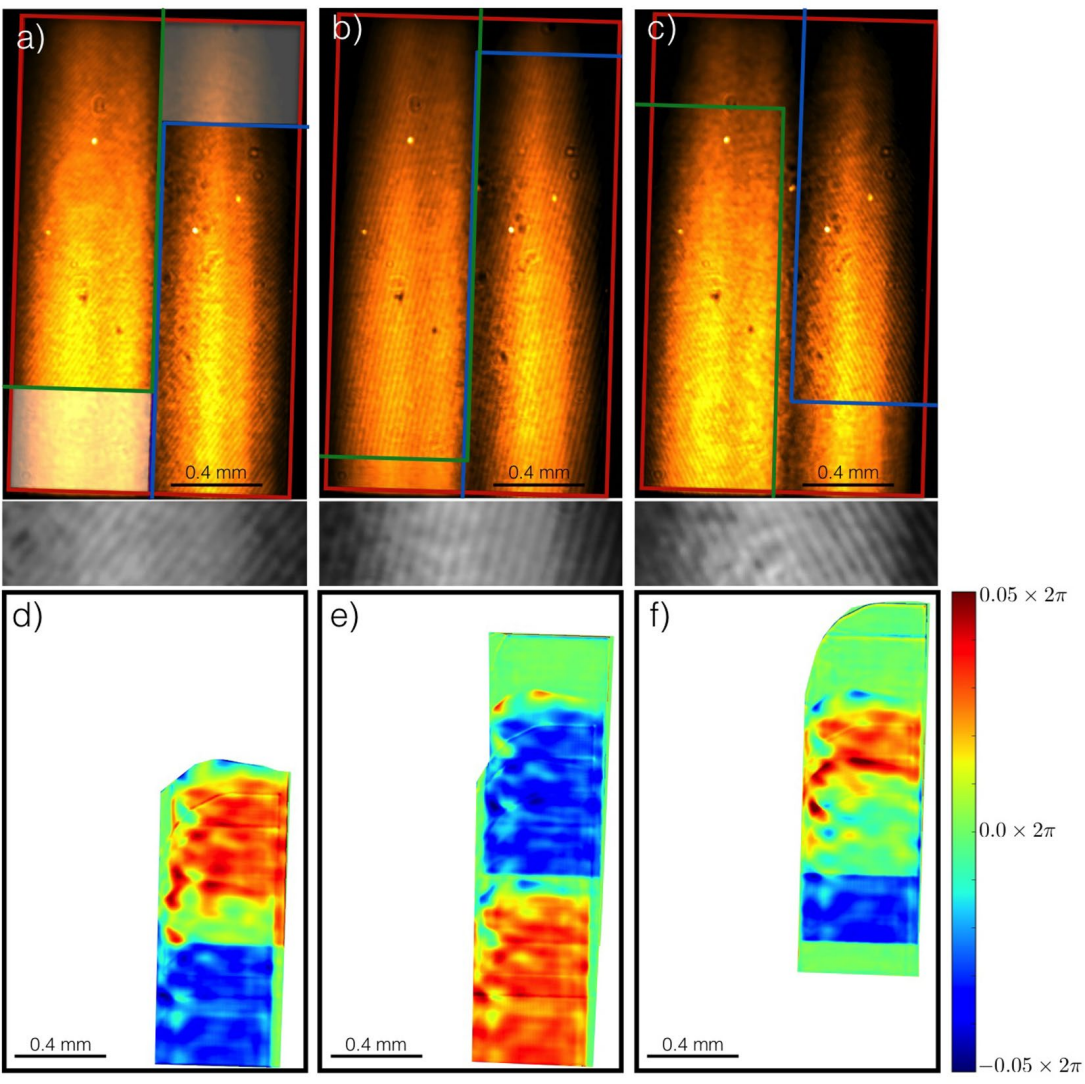
Top (**a**–**c**): the three ronchigrams used to calculate the phase of the X-ray beam, with a magnified close-up of the fringes below. The images are taken with gratings with the different spatial periods (225 nm for (**a**,**c**), and 275 nm for (**b**)), at the same position with respect to the focus, but with an angle of the grating with respect to the vertical of −37.9° for (**a**), −15.4° for (**b**) and 29.6° for (**c**). The red rectangle is the analysis mask of the zeroth order, the green and blue rectangles are the position of the −1 and +1 orders respectively. There is no interference and hence no phase information in the white shade area in (**a**). Bottom (**d**–**f**): False-color image of the difference (i.e. errors) between the phase derived from the Ronchigrams, and the phase derived after re-shearing the recovered wavefront of the beam. RMS error of the images is *λ*/55 for (**a**) and *λ*/40 for (**b**,**c**).

The Ronchi target functions as a diffraction grating for the incoming, focused X-rays. The spatial frequency of the grating is chosen such that the first orders overlap with the fundamental, but do not overlap with each other. The best configuration is attained when the diffraction angle is half of the full-angle divergence of the focused X-ray beam. Indeed, for a larger diffraction angle the overlap is less, and there is a central part of the beam for which there is no interference data available, and therefore the phase will not be determined by the measurement in this area. On the other hand, if the diffraction angle is too small, the +1 and −1 orders will overlap, interfere with each other and not only with the zeroth order, and the phase recovery method will not work. When the spatial period of the grating equals 2*f*_#_ *λ*, with *f*_#_ the f-number of the optic and *λ* the wavelength of the X-rays, the ideal overlap between the first orders and the zeroth order is attained. The zeroth and first order interfere and cause a fringe pattern on the detector. Figure 1 shows that this pattern arises from the interference of two sources that emit spherical wavefronts: the focus of the zeroth order, and the (virtual) focus of the first order. The fringes are therefore analogous to those of Young’s double slit experiment, and it can easily be shown that the phase difference between the zeroth order and first order changes linearly with *x* according to

1 $${\rm{\Delta }}\phi =2\pi \frac{{z}_{1}}{D{z}_{2}}x+{C}_{x}$$

with *z*_2_ the distance between the X-ray camera and the focus, *z*_1_ the distance between the Ronchi grating and the focus, *D* the period of the grating, and *C*_*x*_ a constant in *x* (see methods section for more details). The phase difference results in a linear fringe pattern on the detector, called Ronchigrams. Three Ronchigrams of the beam are seen in Fig. 2(a–c), where the analysis mask of the zeroth order is shown by the red rectangle, and the positions of the +1 and −1 orders by the blue and green rectangles respectively. The fringe density can be tuned to the resolution of the camera by translating the grating with respect to the focus, which effectively changes *z*_1_ in Eq. (1).

As can be seen in Fig. 2 the X-ray beam on the camera looks very asymmetrical, and has two big vertical lobes. The two lobes are caused by the fact the beam overfills a steering mirror located approximately 330 m upstream of the CXI endstation, causing this feature in the far field image. The total beam size in the vertical direction is roughly 70% bigger than in the horizontal direction. This is caused by the fact that the vertical KB mirror has a smaller focal length and therefore sits closer to the focus (500 mm vs 900 mm), leading to a higher divergence and hence a larger vertical size on the detector.

Aberrations in the wavefront of the focused beam will result in a distortion of the fringe pattern. Standard Fourier transform and phase unwrapping methods^25^ are used to calculate the phase of the Ronchigrams. However, the retrieved phase is not the phase of the X-ray beam itself, but the phase difference between the beam and a shifted copy of itself. The Ronchi test is a shearing interferometer and quantitative analysis requires one to invert the shearing operator. Shearing interferometry is well described in the literature and many methods exist to analyse the interferograms^26–30^. The main difficulty stems from the fact that the shearing operator has a kernel, and therefore cannot be inverted mathematically. Indeed, if we define the shearing operator in the x-direction as:

2 $${\hat{S}}_{x}[f(x,y)]=f(x,y)-f(x-{s}_{x},y)$$

we see that any periodic function in *x* with a period *s*_*x*_ gets mapped onto the null vector. Therefore, trying to determine the beam that gives rise to the Ronchigrams is a mathematically ill-posed problem^31^. The most common way to resolve this issue is by using standard regularization theory^32^. In this paper we will follow that road, and adapt an algorithm described in Servin *et al*.^33^, in which two orthogonal interferograms are used in combination with an *a priori* assumption of smoothness of the wavefront. However, an additional difficulty with the Ronchigrams is that the shear is half the beam-size which is large in comparison to typical shearograms. This can result in a large area of the beam where no shearing information is available. For example, in Fig. 2(a) we have no shearing information and therefore no information on the phase in the white shaded area, which is approximately a quarter of the total beam aperture. Furthermore, the area where we do see fringes only yields information on the phase differences in one direction: we do not have any information on phase changes in the beam parallel to the fringes. This problem can be overcome by using enough Ronchigrams to ensure that every area where there is appreciable beam intensity is sheared along at least two angles. In the results presented here, we use the three Ronchigrams shown in Fig. 2(a–c). We only use the interference between the fundamental and the +1 order (i.e. right side of the image) since the interference with the −1 yields exactly the same phase information. Together, the three Ronchigrams contain enough information to retrieve the phase of the whole beam since they ensure we have shearograms in at least two direction in almost the entire aperture. An added bonus is that the three shearograms effectively shear both the horizontal and vertical directions with two incommensurate shear values and since Jacobi has shown that a double periodic function with incommensurate periods is necessarily constant^34^, this removes nearly the whole kernel. Common to interferometric methods, the implicit assumption is made that the beam is spatially coherent, and hence only the coherent part of the beam will be measured. A full description of the inversion algorithm can be found in the methods section of this paper.

## Results

To validate the inversion, we have sheared the recovered wavefront at angles −37.9°, −15.4° and 29.6° and compared them to the measured phase of the Ronchigrams. The result is shown in Fig. 2(d–f). We find an RMS error of less than $\tfrac{1}{40}$ of a wavelength for each Ronchigram, ensuring that the inversion algorithm works accurately. We note that the use of three Ronchigrams over-constrains the optimization problem. Indeed, any two Ronchigrams with different shearing directions can always be used to invert the shearing operator with a vanishing error. However, this is not the case when three or more shearograms are used: only those derived from a physical field will result in an illumination phase that yields a small RMS error between the measured shearograms and the calculated ones. This is important since we use three shearograms from three different FEL pulses, and we make the implicit assumption that the phase of these pulses are the same. The fact that such a small RMS error is found validates this assumption and the method in general.

**Figure 3:**
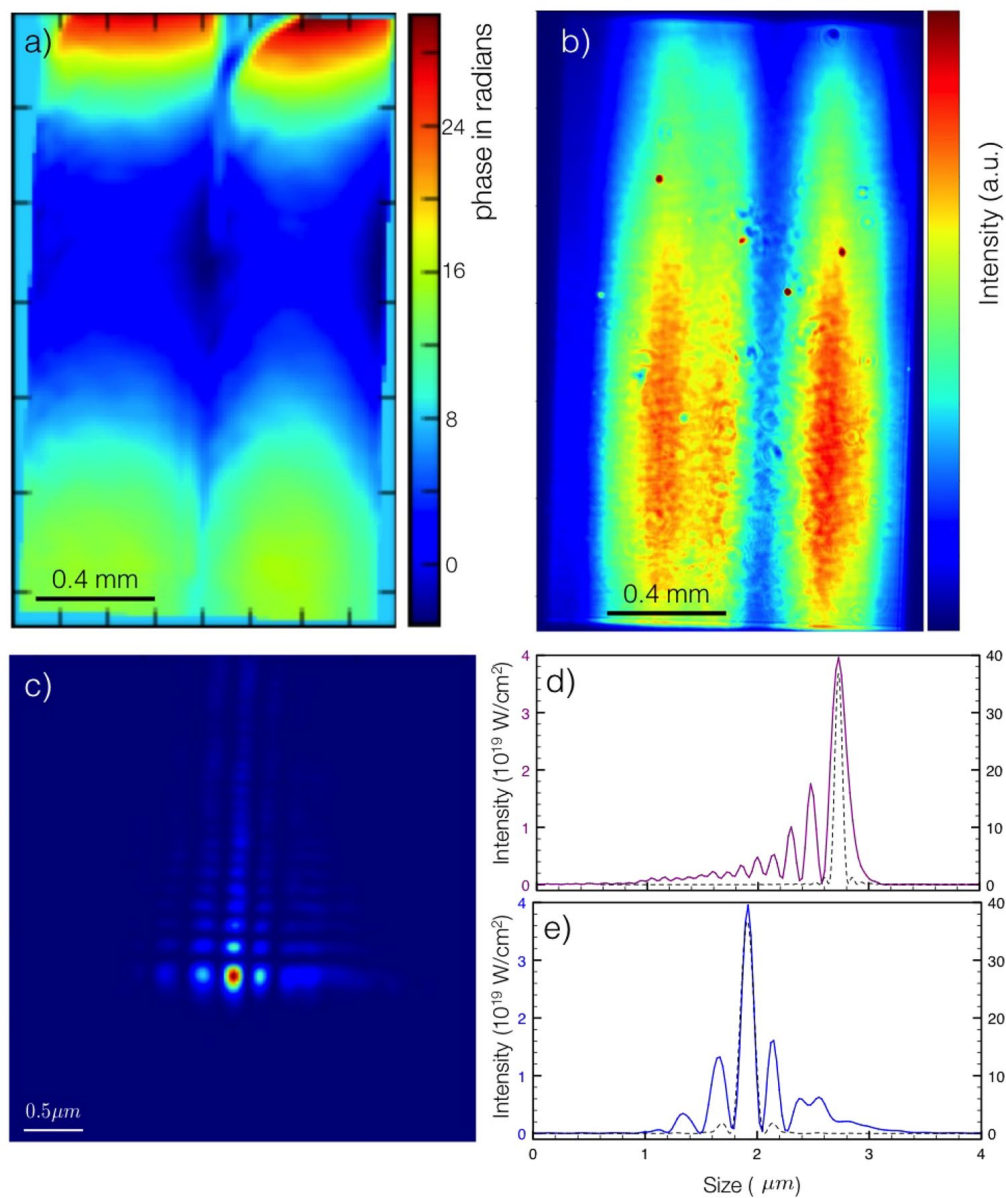
(**a**) Wavefront of the x-ray beam, (**b**) Measured intensity of the beam. (**c**) Intensity of the focal spot at best focus. (**d**) Vertical lineout (red) and (**e**) horizontal lineout (blue) through focus, compared with the theoretical ideal focus (dashed) when no abberations would be present. The peak intensity is 3.9 10^19^ W/cm^2^ for the 3 mJ beam energy and 60 fs pulse length that was used in the experiment.

The recovered phase can be seen in Fig. 3(a), while Fig. 3(b) shows the measured intensity of the beam. Using this phase and measured intensity profile, we can calculate the X-ray profile at focus, which is shown in Fig. 3(c–e). The full width at half maximum of focal size of the central peak is 167 nm in the vertical and 123 nm in the horizontal. The uncertainty in the wavefront measurement of *λ*/40 mentioned above results in an uncertainty of approximately 2% in the peak intensity of the beam, applying the Strehl Ratio/Ruze formula^35,36^.

## Discussion

**Figure 4:**
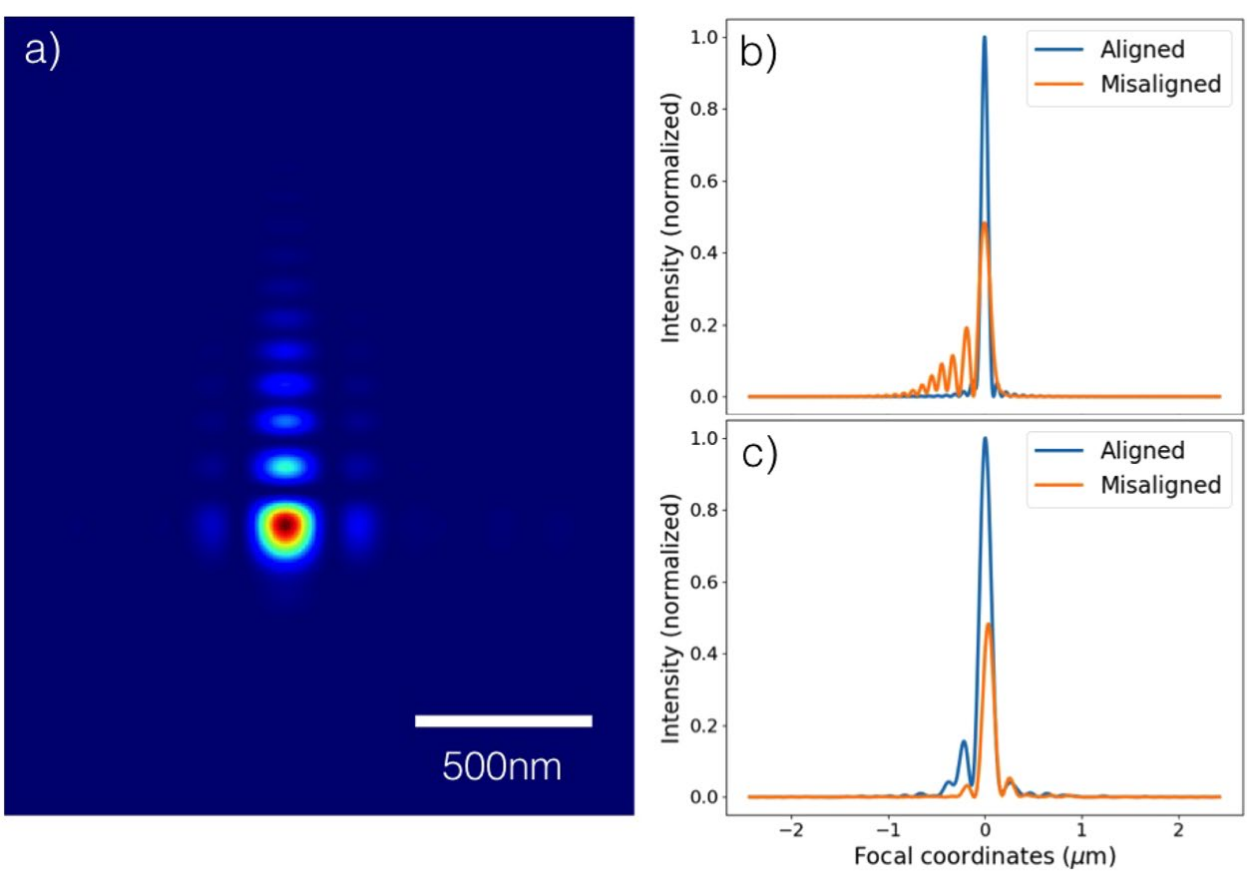
(**a**) Simulation of the focus of the focal spots of a misaligned KB-pair. The vertical focusing mirror was misaligned by 9 *μ*rad and the horizontal focusing mirror by 7.5 *μ*rad. (**b**) Vertical lineout of the spot in (**a**), compared with the spot from a perfectly aligned mirror. (**c**) Horizontal lineout.

In order to demonstrate the value of the information gained from the Ronchigram wavefront retrieval, a simulation of the aberrations due to misalignment of the KB mirror pair was performed. In the simulation, the vertical focusing mirror (VFM) was misaligned by 9 *μ*rad and the horizontal focusing mirror (HFM) was misaligned by 7.5 *μ*rad, resulting in a best focus 2 mm upstream of the nominal focus position. Horizontal and vertical lineouts are shown in Fig. 4, along with lineouts corresponding to operation of the system with the mirrors aligned to the design angle. From comparison with Fig. 3(d,e), it is clear that the majority of the observed vertical aberrations are captured in the simulated misalignment of the VFM. However, the horizontal direction is strongly affected by aberrations caused by two horizontal steering mirrors 330 m upstream of the KB pair. While an attempt was made to account for these aberrations in the simulation, the Ronchigram result suggests that there are additional aberrations that were not fully captured in the simulation. The analysis presented here underscores the importance of techniques which retrieve both the amplitude and phase of the focus. Other techniques, such as the use of imprints, rely on the assumption that the interaction plane has been chosen correctly. As can be seen in Fig. 4, a small error of 2 mm along the beam axis can result in a peak intensity that is a factor of two below what can be achieved with ideal alignment.

In conclusion, we have presented a method to determine the focus of Free electron laser using Ronchi shearing interferometry. The method is fast, *in situ* and does not require high beam pointing stability. The method has been applied to the nanofocus of the CXI beamline at LCLS, and is readily applicable to other X-ray beamlines and focal sizes.

## Methods

### Ronchigrams

A Ronchi grating with a duty cycle of 1 and with the orientation of the grating in the *x* direction can be described as:3where $\bigstar$ denotes the convolution operator, *T*_1_ and *T*_2_ are the complex transmission functions the two parts of the grating, the Dirac comb  is defined as4and the rectangle function defined as

5 $${{\rm{rect}}}_{L}(x)=\{\begin{array}{ll}1 & {\rm{if}}\,-L\mathrm{/2} < x < L\mathrm{/2}\,\\ 0 & {\rm{elsewhere}}\,\end{array}$$

Taking the two dimensional Fourier transform of the Ronchi grating we get:

6 $$ {\mathcal R} ({k}_{x},{k}_{y})=2\pi \,\sum _{l=-\infty }^{+\infty }\,{R}_{l}\delta ({k}_{x}-{k}_{l})\,\delta ({k}_{y})$$

with

7 $${k}_{l}=\frac{2\pi }{D}l\,{\rm{with}}\,l\in {\mathbb{Z}}$$

8 $${R}_{l}=\{\begin{array}{ll}({T}_{1}+{T}_{2})\pi  & {\rm{if}}\,l=0\\ ({T}_{2}-{T}_{1})\pi \,\sin {\rm{c}}(\frac{l\pi }{2}) & {\rm{if}}\,l\in {{\mathbb{Z}}}_{0}\end{array}$$

with sinc(*x*) = sin (*x*)/*x* and using the the two-dimensional Fourier transform defined as:

9 $$F({k}_{x},{k}_{y})= {\mathcal F} [f(x,y)]={\int }_{-\infty }^{\infty }\,f(x,y){e}^{-i({k}_{x}x+{k}_{y}y)}dx$$

**Figure 5:**
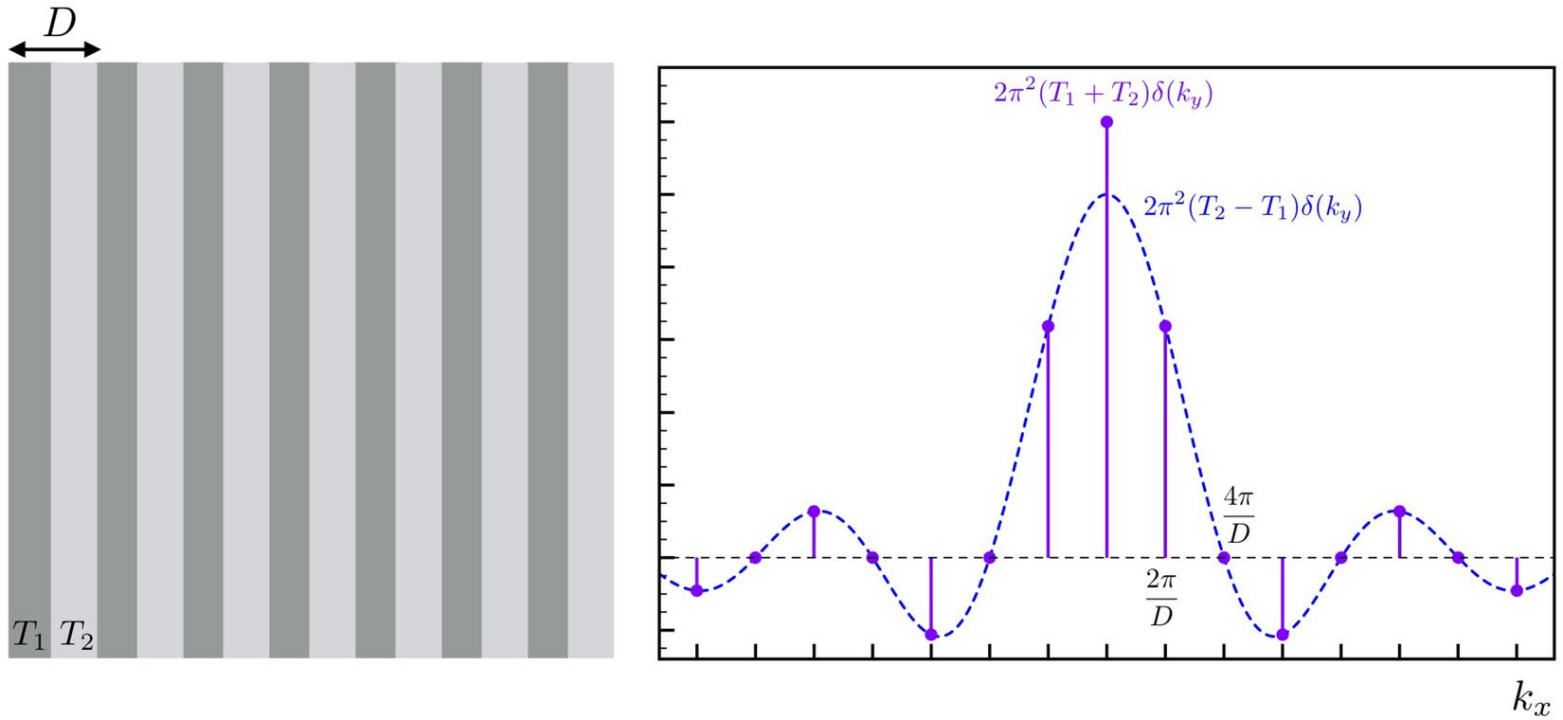
The Ronchi target (left) and its 2D Fourier transform. *T*_1_ and *T*_2_ are complex transmission functions. The dots in the Fourier transform plot signify delta-distributions.

The Ronchi grating and its Fourier transform can be seen in Fig. 5. We now consider the X-ray beam with a focus located at *z* = 0, with electric field *E*_0_(*x*, *y*, 0). Using the paraxial approximation, we can propagate the electric field to the camera position, *z*_*c*_, using the Fresnel intergral:

10 $${E}_{c0}(x,y)={P}_{{z}_{c}}(x,y)\,{E}_{c0}^{F}(x,y)$$

with

11 $${E}_{c0}^{F}(x,y)=-\frac{ik}{2\pi {z}_{c}}{e}^{ik{z}_{c}} {\mathcal F} \,{[{E}_{0}(x,y){P}_{{z}_{c}}(x,y)]}_{\begin{array}{c}{k}_{x}=\tfrac{k\cdot x}{{z}_{c}}\\ {k}_{y}=\tfrac{k\cdot y}{{z}_{c}}\end{array}}$$

with *k* the wavenumber of the electromagnetic field and the spherical phase factor ${P}_{{z}_{c}}$ defined as:

12 $${P}_{{z}_{c}}(x,y)=\exp \,(\frac{ik}{2{z}_{c}}({x}^{2}+{y}^{2}))$$

Basically, ${E}_{c0}^{F}$ is the electric field at the camera position without the spherical wavefront curvature due to the propagation distance *z*_*c*_. We now place the Ronchi grating at position *z* = *z*_1_, and propagate the beam from focus to the grating using the Fresnel integral. We multiply the field with the transmission function of the Ronchi grating and then propagate the field back to the (now virtual) focus of the beam. We get the resulting (virtual) field *E*_*R*0_:

13 $${E}_{R0}=\frac{1}{2\pi }\,\sum _{l=-\infty }^{+\infty }\,{R}_{l}\,\exp \,(i\frac{{k}_{l}{X}_{l}}{2})\,{E}_{0}(x+{X}_{l},y,\mathrm{0)}\,\exp \,(i{k}_{l}x)$$

with

14 $${X}_{l}=\frac{2\pi {z}_{1}}{kD}l\,{\rm{with}}\,l\in {\mathbb{Z}}$$

We use the Fresnel integral to propagate this field to *z* = *z*_*c*_. Substituting equation (11) we get:

15 $${E}_{Rc}=\frac{1}{2\pi }{P}_{{z}_{c}}(x,y)\,\sum _{l=-\infty }^{+\infty }\,{R}_{l}{e}^{i{\phi }_{l}}\,{E}_{c0}^{F}(x-{X}_{D}^{l},y,{z}_{c})\,\exp \,(i{k}_{l}\frac{{z}_{1}}{{z}_{c}}x)$$

with

16 $${\phi }_{l}=\frac{{k}_{l}{X}_{l}}{2}\,(\frac{{z}_{1}}{{z}_{c}}-1)$$

17 $${X}_{D}^{l}=\frac{{k}_{l}}{k}({z}_{c}-{z}_{1})$$

When different orders (i.e. different values of *l*) overlap, they will interfere, and form predominant linear fringes due to the linear phase in x. For our ronchi test, we choose *D* to have the half-beam overlap as shown in Fig. 2. From equation (15) we can calculate the phase difference between the zeroth and first order:

18 $${\rm{\Delta }}\phi ={\phi }_{{R}_{0}}-{\phi }_{{R}_{1}}-\frac{2{\pi }^{2}{z}_{1}}{k{D}^{2}}\,(\frac{{z}_{1}}{{z}_{c}}-1)+{\hat{S}}_{{X}_{D}}[{\phi }_{c0}(x,y)]-\frac{2\pi {z}_{1}}{{z}_{c}D}x$$

with ${\phi }_{{R}_{0}}$ the phase of *R*_0_, ${\phi }_{{R}_{1}}$ the phase of *R*_1_, *ϕ*_*c*0_(*x*, *y*) the phase of ${E}_{c0}^{F}(x,y,{z}_{c})$, and ${\hat{S}}_{{X}_{D}}$ the shearing operator defined as:

19 $${\hat{S}}_{{X}_{D}}[f(x,y)]=f(x,y)-f(x-{X}_{D},y)$$

and ${X}_{D}\equiv {X}_{D}^{1}$. The first three terms give the constant phase difference between the orders (corresponding to the undetermined *C*_*x*_ in equation (1). This constant phase is a priory unknown, since we cannot know the exact position of the beam with respect to the grating, and a shift of *δx* in this position will lead to a constant phase of $2\pi \tfrac{\delta x}{D}$. The value of this constant phase is actually important during the shear-inversion, and will need to be optimized together with the rest of the wavefront. It could also be used to measure beam jitter, if it is not larger than the grating period. The last two terms show how the phase varies in *x* and *y*. The linear phase in *x* of the last term will result in linear fringes in the intensity of *E*_*RC*_, provided the spatial frequency $\frac{2\pi {z}_{1}}{{z}_{c}D}$ is large enough. Using standard Fourier methods^25^ and phase unwrapping algorithms we can retrieve ${\hat{S}}_{{X}_{D}}[{\phi }_{c0}(x,y)]$ as long as the spatial frequency $\frac{2\pi {z}_{1}}{{z}_{c}D}$ is at least twice the highest spatial frequency that is present in the intensity of *E*_*RC*_; otherwise aliasing will occur. To retrieve the phase of the electric field at the camera location *ϕ*_*co*_(*x*, *y*), we will have to invert the shearing operator ${\hat{S}}_{{X}_{D}}$. Measuring the intensity of *E*_*c*0_ is trivially done without Ronchi grating. Therefore, we will have full information of both phase and amplitude of the electric field at the camera location, which allows us to propagate the beam to any *z* location, and therefore fully determine its focal characteristics.

### Inverting the shearing operator

As shown above, the phase retrieved from the Ronchigrams is not the actual phase of the X-ray beam, but the sheared phase. In general we have:

20 $${\hat{S}}_{\bar{s}}[\phi (x,y)]=\phi (x,y)-\phi (x-{s}_{x},y-{s}_{y})$$

with the shear vector $\bar{s}=({s}_{x},{s}_{y})$ orthogonal to the lines of the Ronchi grating. The measured data will be sampled in *x* and *y*, and defining *ϕ*_*i*,*j*_ as the phase at the sample points (i.e., pixels), we have the corresponding discrete operator

21 $${\hat{S}}_{\bar{s}}[{\phi }_{i,j}]={\phi }_{i,j}-{\phi }_{i-{s}_{x},j-{s}_{y}}$$

with *s*_*x*_ and *s*_*y*_ expressed in number of pixels. We call ${\phi }_{i,j}^{\bar{s}}$ the measured sheared phase retrieved from the Ronchigram. We now search for a solution of *ϕ*_*i*,*j*_ that minimizes the cost function of the mean-square error between the sheared phase and the measured one:

22 $${U}_{\bar{s}}^{2}=\sum _{i,j}\,{({\hat{S}}_{\bar{s}}[{\phi }_{i,j}]-{\phi }_{i,j}^{\bar{s}}+{\phi }_{c}^{\bar{s}})}^{2}\,{P}_{i,j}^{\bar{s}}$$

with ${\phi }_{c}^{\bar{s}}$ the constant phase that is jitter dependent mentioned above and ${P}_{i,j}^{\bar{s}}$ the masking function that is equal to 1 where good sheared data is available and 0 where there isn’t. As mentioned above, we need multiple Ronchigrams to recover the phase, due to the limited overlap between the beams after shearing. Therefore the total cost function will be the sum for different values of the sheared direction $\bar{s}$:

23 $${U}^{2}=\sum _{\bar{s}}{U}_{\bar{s}}^{2}$$

In the reconstruction that is shown in the main body of this paper we use three values of $\bar{s}$, corresponding to shears at −37.9°, −15.4° and 29.6°, but in principle we could use more shearograms and reduce the error. As in Servin *et al*.^33^ we will add a cost function that corresponds to our *a priori* assumption of smoothness in *x* and *y*:

24 $${R}_{x}^{2}=\sum _{i,j}\,{({\phi }_{i-\mathrm{1,}j}-2{\phi }_{i,j}+{\phi }_{i+\mathrm{1,}j})}^{2}\,{P}_{i-\mathrm{1,}j}\,{P}_{i+\mathrm{1,}j}$$

25 $${R}_{y}^{2}=\sum _{i,j}\,{({\phi }_{i,j-1}-2{\phi }_{i,j}+{\phi }_{i,j+1})}^{2}\,{P}_{i,j-1}\,{P}_{i,j+1}$$

where *P*_*i*,*j*_ is masking function equal to 1 inside the aperture of the beam, and 0 outside it. Note that in principle we could use

26 $${P}_{i,j}^{\bar{s}}={P}_{i,j}\,{P}_{i-{s}_{x},j-{s}_{y}}$$

although in practise we may need to take the mask slightly smaller. Alternatively, we could allow values in the masking function between 0 and 1 to allow for a weighted average in in the cost function. With the regularization, the total cost function becomes:

27 $${U}^{2}=\sum _{\bar{s}}\,{U}_{\bar{s}}^{2}+\eta \,({R}_{x}^{2}+{R}_{y}^{2})$$

with *η* the regularization parameter. Efficient minimization of the cost function requires the partial derivatives toward *ϕ*_*i*,*j*_ and ${\phi }_{c}^{\bar{s}}$:

28 $$\frac{\partial {U}^{2}}{\partial {\phi }_{k,l}}=\sum _{\bar{s}}\,\frac{\partial {U}_{\bar{s}}^{2}}{\partial {\phi }_{k,l}}+\eta \,(\frac{\partial {R}_{x}^{2}}{\partial {\phi }_{k,l}}+\frac{\partial {R}_{y}^{2}}{\partial {\phi }_{k,l}})$$

29 $$\frac{\partial {U}^{2}}{\partial {\phi }_{c}^{\bar{s}}}=\frac{\partial {U}_{\bar{s}}^{2}}{\partial {\phi }_{c}^{\bar{s}}}$$

with

30 $$\begin{array}{rcl}\frac{\partial {U}_{\bar{s}}^{2}}{\partial {\phi }_{k,l}} & = & 2{P}_{k,l}^{\bar{s}}({\hat{S}}_{\bar{s}}[{\phi }_{k,l}]-{\phi }_{k,l}^{\bar{s}}+{\phi }_{c}^{\bar{s}})\\  &  & -2{P}_{k+{s}_{x},l+{s}_{y}}^{\bar{s}}({\hat{S}}_{\bar{s}}[{\phi }_{k+{s}_{x},l+{s}_{y}}]-{\phi }_{k+{s}_{x},l+{s}_{y}}^{\bar{s}}+{\phi }_{c}^{\bar{s}})\end{array}$$

31 $$\frac{\partial {U}_{\bar{s}}^{2}}{\partial {\phi }_{c}^{\bar{s}}}=2\,\sum _{k,l}\,{P}_{k,l}^{\bar{s}}({\hat{S}}_{\bar{s}}[{\phi }_{k,l}]-{\phi }_{k,l}^{\bar{s}}+{\phi }_{c}^{\bar{s}})$$

32 $$\begin{array}{rcl}\frac{\partial {R}_{x}^{2}}{\partial {\phi }_{k,l}} & = & 2({\phi }_{k,l}-2{\phi }_{k+\mathrm{1,}l}+{\phi }_{k+\mathrm{2,}l})\,{P}_{k,l}\,{P}_{k+\mathrm{2,}l}\\  &  & -\,4({\phi }_{k-\mathrm{1,}l}-2{\phi }_{k,l}+{\phi }_{k+\mathrm{1,}l})\,{P}_{k-\mathrm{1,}l}\,{P}_{k+\mathrm{1,}l}\\  &  & +\,2({\phi }_{k-\mathrm{2,}l}-2{\phi }_{k-\mathrm{1,}l}+{\phi }_{k,l})\,{P}_{k-\mathrm{2,}l}\,{P}_{k,l}\end{array}$$

33 $$\begin{array}{rcl}\frac{\partial {R}_{y}^{2}}{\partial {\phi }_{k,l}} & = & 2({\phi }_{k,l}-2{\phi }_{k,l+1}+{\phi }_{k,l+2})\,{P}_{k,l}\,{P}_{k,l+2}\\  &  & -\,4({\phi }_{k,l-1}-2{\phi }_{k,l}+{\phi }_{k,l+1})\,{P}_{k,l-1}\,{P}_{k,l+1}\\  &  & +\,2({\phi }_{k,l-2}-2{\phi }_{k,l-1}+{\phi }_{k,l})\,{P}_{k,l-2}\,{P}_{k,l}\end{array}$$

We can now minimize equation (27) using a conjugate gradient descent method; alternatively a limited memory Broyden-Fletcher-Goldfard-Shanno algorithm^37–41^ runs very fast.

## References

[1] Ackermann W (2007). Operation of a free-electron laser from the extreme ultraviolet to the water window. Nature Photon..

[2] Allaria E (2015). The FERMI free-electron lasers. J. Synchrotron Rad..

[3] Emma P (2010). First lasing and operation of an å ngstrom-wavelength free-electron laser. Nature Photon..

[4] Ishikawa T (2012). A compact X-ray free-electron laser emitting in the sub-angstrom region. Nature Photon..

[5] Tschentscher T (2017). Photon beam transport and scientific instruments at the european xfel. Applied Sciences.

[6] Bostedt C (2016). Linac coherent light source: The first five years. Rev. Mod. Phys..

[7] Schlichting I, White WE, Yabashi M (2015). An introduction to the special issue on X-ray free-electron lasers. J. Synchrotron Rad..

[8] Seibert MM (2011). Single mimivirus particles intercepted and imaged with an x-ray laser. Nature.

[9] Chalupský J (2015). Imprinting a focused x-ray laser beam to measure its full spatial characteristics. Phys. Rev. Applied.

[10] Schropp A (2013). Full spatial characterization of a nanofocused x-ray free-electron laser beam by ptychographic imaging. Scientific reports.

[11] Kayser Y (2014). Wavefront metrology measurements at sacla by means of x-ray grating interferometry. Opt. Express.

[12] Weitkamp T, Nhammer B, Diaz A, David C, Ziegler E (2005). X-ray wavefront analysis and optics characterization with a grating interferometer. Applied Physics Letters.

[13] Assoufid L (2016). Development and implementation of a portable grating interferometer system as a standard tool for testing optics at the advanced photon source beamline 1-bm. Review of Scientific Instruments.

[14] Ronchi V (1964). Forty years of history of a grating interferometer. Appl. Opt..

[15] Uhlén F (2014). Ronchi test for characterization of X-ray nanofocusing optics and beamlines. J. Synchrotron Rad..

[16] Nilsson D (2012). Ronchi test for characterization of nanofocusing optics at a hard x-ray free-electron laser. Opt. Lett..

[17] Liang M (2015). The coherent x-ray imaging instrument at the linac coherent light source. J. Synchrotron Rad..

[18] Boutet S, Williams GJ (2010). The coherent x-ray imaging (cxi) instrument at the linac coherent light source (lcls). New Journal of Physics.

[19] Siewert F (2012). Ultra-precise characterization of lcls hard xray focusing mirrors by high resolution slope measuring deflectometry. Opt. Express.

[20] Soufli, R. *et al*. Morphology, microstructure, stress and damage properties of thin film coatings for the lcls x-ray mirrors morphology, microstructure, stress and damage properties of thin film coatings for the lcls x-ray mirrors. In Juha, L., Bajt, S. & Sobierajski, R. (eds) *Damage to VUV*, *EUV*, *and X*-*Ray Optics II*, vol. 7361 of *Damage to VUV*, *EUV*, *and X*-*Ray Optics II*, 73610U–1. SPIE (Proc. of SPIE, 2009).

[21] Barty A (2009). Predicting the coherent x-ray wavefront focal properties at the linac coherent light source (lcls) x-ray free electron laser. Opt. Express.

[22] Hoszowska, J. *et al*. X-ray two-photon absorption with high fluence xfel pulses. In *XXIX International Conference on Photonic*, *Electronic*, *and Atomic Collisions* (*ICPEAC2015*), vol. 635 of *Journal of Physics*: *Conference Series*, 102009 (IOP Publishing, 2015).

[23] Fuchs M (2015). Anomalous nonlinear x-ray compton scattering. Nature Phys..

[24] Nass K (2015). Indications of radiation damage in ferredoxin microcrystals using high-intensity x-fel beams. J. Synchrotron Rad..

[25] Takeda M, Ina H, Kobayashi S (1982). Fourier-transform method of fringe-pattern analysis for computer-based topography and interferometry. J. Opt. Soc. Am..

[26] Elster C (2000). Exact two-dimensional wave-front reconstruction from lateral shearing interferograms with large shears. Appl. Opt..

[27] Elster C, Weingärtner I (1999). Solution to the shearing problem. Appl. Opt..

[28] Elster C (1999). Recovering wavefronts from difference measurements in lateral shearing interferometry. Journal of Computational and Applied Mathematics.

[29] Elster C, Weingärtner I (1999). Exact wave-front reconstruction from two lateral shearing interferograms. J. Opt. Soc. Am. A.

[30] Liang P, Ding J, Jin Z, Guo C-S, tian Wang H (2006). Two-dimensional wave-front reconstruction from lateral shearing interferograms. Opt. Express.

[31] Hadamar J (1902). Sur les problèmes aux dérivées partielles et leur signification physique. Princeton University Bulletin.

[32] Tikhonov AN (1963). Solution of incorrectly formulated problems and the regularization method. Sov. Math. Dokl..

[33] Servin M, Malacara D, Marroquin JL (1996). Wave-front recovery from two orthogonal sheared interferometers. Appl. Optics.

[34] Jacobi CGJ (1835). De functionibus duarum variabilium quadrupliciter periodicis, quibus theoria transcendentium abelianarum innitur. J. für Math..

[35] Mahajan VN (1983). Strehl ratio for primary aberrations in terms of their aberration variance. J. Opt. Soc. Am..

[36] Ruze J (1952). The effect of aperture errors on the antenna radiation pattern. Il Nuovo Cimento.

[37] Broyden CG (1970). The convergence of a class of double-rank minimization algorithms 1. general considerations. IMA Journal of Applied Mathematics.

[38] Fletcher R (1970). A new approach to variable metric algorithms. The Computer Journal.

[39] Goldfarb D (1970). A family of variable-metric methods derived by variational means. Math. Comp..

[40] Shanno DF (1970). Conditioning of quasi-newton methods for function minimization. Math.Comp..

[41] Nocedal J (1980). Updating quasi-newton matrices with limited storage. Math. Comp..

